# Enhancing Jujube Forest Growth Estimation and Disease Detection Using a Novel Diffusion-Transformer Architecture

**DOI:** 10.3390/plants13172348

**Published:** 2024-08-23

**Authors:** Xiangyi Hu, Zhihao Zhang, Liping Zheng, Tailai Chen, Chao Peng, Yilin Wang, Ruiheng Li, Xinyang Lv, Shuo Yan

**Affiliations:** China Agricultural University, Beijing 100083, China

**Keywords:** growth estimation, disease detection, plant image analysis, model generalization in complex environments, diffusion-transformer architecture

## Abstract

This paper proposes an advanced deep learning model that integrates the Diffusion-Transformer structure and parallel attention mechanism for the tasks of growth estimation and disease detection in jujube forests. Existing methods in forestry monitoring often fall short in meeting the practical needs of large-scale and highly complex forest areas due to limitations in data processing capabilities and feature extraction precision. In response to this challenge, this paper designs and conducts a series of benchmark tests and ablation experiments to systematically evaluate and verify the performance of the proposed model across key performance metrics such as precision, recall, accuracy, and F1-score. Experimental results demonstrate that compared to traditional machine learning models like Support Vector Machines and Random Forests, as well as common deep learning models such as AlexNet and ResNet, the model proposed in this paper achieves a precision of 95%, a recall of 92%, an accuracy of 93%, and an F1-score of 94% in the task of disease detection in jujube forests, showing similarly superior performance in growth estimation tasks as well. Furthermore, ablation experiments with different attention mechanisms and loss functions further validate the effectiveness of parallel attention and parallel loss function in enhancing the overall performance of the model. These research findings not only provide a new technical path for forestry disease monitoring and health assessment but also contribute rich theoretical and experimental foundations for related fields.

## 1. Introduction

In global agricultural and forestry management, plant health status and disease detection have been key research areas [1]. Particularly in Northern China, jujube serve as an important economic crop, and their growth condition and disease prevention directly impact local farmers’ income and ecological balance [2,3]. Traditional methods for plant disease detection, relying on manual inspection and expert judgment, are not only time-consuming and labor-intensive but also prone to inaccuracies due to subjective human factors [4,5,6]. In recent years, with the development of remote sensing technology and deep learning [7], image-based plant health assessment and disease detection methods have increasingly become a research focus [8,9].

Advancements in computer vision have led to the widespread application of image recognition technologies in agricultural disease detection [10]. By analyzing images captured continuously during the crop’s growing period, computer vision models are capable of assessing the crop’s growth rate [11] and predicting its final yield. Image regression techniques allow these models to read yield predictions during the crop’s growth phase, enhancing crop yield and quality through scientific management. Acharya, Snehaprava et al. [12] used Support Vector Machine (SVM) and its variant, Least Square SVM (LS-SVM), to detect early rice diseases. Experimental results showed that LS-SVM outperformed SVM in terms of accuracy. Ahmed Imtiaz et al. [13] developed plant disease detection models using machine learning methods such as Random Forest, Linear Regression, and Naive Bayes. However, these methods performed poorly when there were missing data or large feature sets.

With the development of deep learning, computer vision technology has a profound impact on agricultural economics, as accurate disease identification and classification technologies enable agricultural producers to promptly detect and address crop diseases, reducing pesticide use, lowering environmental pollution, and minimizing economic losses [14]. Wakhare, Prashant B et al. [15] used the AlexNet algorithm to detect two major diseases, bacterial blight and Alternaria leaf spot. They created a dataset of a total of 1245 pomegranate leaf images, and the results showed an accuracy rate of up to 97.60%. Archana U et al. [16] conducted disease classification based on tomato leaves. The results produced by ResNet-50 showed significant results, with an accuracy rate of 96.35%. Genze et al. [17] further expanded the dataset in size and variety, utilizing a convolutional neural network (CNN) architecture to achieve high mean average precision (mAP) of approximately 97.9%, 94.2%, and 94.3% on retention test datasets for corn, rye, and witchgrass, respectively.

Guo et al. [18] utilized a Region Proposal Network (RPN) to identify and locate leaves in complex environments, then employed the Chan–Vese (CV) algorithm, based on the results of the RPN, to segment images containing symptomatic features; finally, segmented leaves were input into a transfer learning model trained on a dataset of diseased leaves against simple backgrounds. This method achieved an accuracy rate of 83.57%. Su et al. [19] constructed a bandpass filter-based fluorescence macro-imaging system for safety testing of celery solutions. Patel Krishna et al. [20], by comparing manual methods and computer vision applications to assess mango attributes, developed various Multiple Linear Regression (MLR) models with accuracies exceeding 97.9%, 93.5%, and 92.5% respectively; however, their use of a monochrome camera was not suitable for broad applications. Alencastre et al. [21], addressing the limitations of monochrome images for use with modern smartphones, utilized a color image dataset and a convolutional neural network for sugarcane quality detection, doubling the performance for the L 01-299 variety and increasing the performance fivefold for the HoCP 09-804 variety, though the dataset was too small.

Building on this, Li et al. [22] further improved by proposing a CNN-based model, which, after training and validation, achieved the best training and validation accuracies of 99% and 98.98%, respectively. Additionally, there is significant potential in crop growth research; Savarimuthu, Nickolas et al. [23] explored the potential of computer vision-based object detection methods for early detection of plant diseases, conducting comparative studies using three different benchmark object detection models: YOLOv4, EfficientDet, and Scaled-YOLOV4. The findings indicate that EfficientDet’s performance is inferior to that of Scaled-YOLOV4; Roy [24], addressing real-time detection of agricultural growth stages, proposed an improved version of the YOLOv4 algorithm framework, Dense-YOLOv4, for real-time detection of mangoes at different growth stages. The proposed model was optimized in terms of detection speed and accuracy. Mandal [25] utilized dual-polarized Sentinel-1 SAR data to derive a new radar vegetation index, called the Dual-polarized Radar Vegetation Index (DpRVI), for monitoring crop growth.

Despite these state-of-the-art deep learning models achieving high-performance results, they consume substantial computational resources for training. To overcome the limitations of these models, numerous studies have emerged, primarily divided into hybrid models and pure Transformer enhancements [26]. For hybrid models, researchers have attempted to combine Transformers and CNNs to enhance performance. For instance, Lu et al. [27] proposed the GeT—Ghost heuristic Transformer model, which builds intermediate feature maps through linear operations based on a CNN. The model achieved an accuracy of 98.14% on a self-collected dataset of grape leaves containing 12,615 images. Similarly, Thakur et al. [28] introduced PlantXViT, consisting of a VGG16 network, an initial block, and Transformer encoder layers. It effectively captures local features of images through the VGG16 network and the initial block, outperforming state-of-the-art CNN models. Many other studies have integrated different CNN blocks into Transformer architectures to enhance feature extraction capabilities [28].

In this study, a jujube forest growth assessment and disease detection system based on the Diffusion-Transformer architecture was developed. This system integrates the latest deep learning technologies tailored specifically to meet practical agricultural needs and address particular challenges in forestry disease detection. The diffusion model, as an emerging generative model technology, simulates the reverse diffusion process during data generation, progressively constructing meaningful data samples from random noise, and demonstrates significant potential in handling complex ecological data and making long-sequence predictions. However, the application of diffusion models in the fields of agriculture and forestry has not yet been widely developed. The potential of applying diffusion models to disease detection and growth assessment in jujube forests was explored, particularly in terms of managing uncertainty and simulating growth processes. The system not only implements an agricultural Transformer structure based on parallel attention but also introduces a parallel loss function, significantly enhancing the efficiency and accuracy of model training and prediction.

Furthermore, the system has been successfully deployed in the Inner Mongolia Bayannur Forestry Bureau, demonstrating superior on-site application capabilities. Through practical deployment, the effectiveness and reliability of the system in actual environmental conditions were verified, providing robust technical support for future forestry management and disease prevention. The development and application of this system mark a significant advancement in the application of deep learning technologies in the agricultural and forestry sectors, laying a solid foundation for future research and development of related technologies.

## 2. Related Work

### 2.1. Diffusion

The diffusion model, originally inspired by the diffusion process in statistical physics, has recently been adopted in the domain of generative models, particularly demonstrating strong capabilities in generating images and audio data [29]. Such models simulate the reverse diffusion process during data generation, progressively constructing meaningful data samples from random noise [30]. Unlike traditional Generative Adversarial Networks (GANs) [31] and Variational Autoencoders (VAEs) [32], diffusion models generate meaningful data samples from random noise through a reverse process, proving effective in handling high-dimensional data and long-sequence data.

The fundamental formula of the diffusion model describes the process where data gradually diffuse from an initial noise distribution to a target data distribution [33]. The forward process of the diffusion model starts from the data distribution $q(x_{0})$, gradually adding noise to form a series of intermediate states $x_{1},x_{2},\ldots ,x_{T}$, until a pure noise distribution $x_{T}$ is reached. The forward diffusion process can be expressed as:

(1) $$q\left(x_{t}|x_{t-1}\right)=N\left(x_{t};\sqrt{\alpha_{t}}x_{t-1},\left(1-\alpha_{t}\right)I\right)$$

where N denotes a normal distribution, and $\alpha_{t}$ is the scaling coefficient at step *t*. The reverse process attempts to revert from $x_{T}$ to $x_{0}$. By learning a parameterized model $p_{\theta }\left(x_{t-1}|x_{t}\right)$, the reverse process gradually removes noise and reconstructs the original data. The reverse diffusion process can be represented as:

(2) $$p_{\theta }\left(x_{t-1}|x_{t}\right)=N\left(x_{t-1};\mu_{\theta }\left(x_{t},t\right),\Sigma_{\theta }\left(x_{t},t\right)\right)$$

where $\mu_{\theta }$ and $\Sigma_{\theta }$ are the parameterized mean and variance to be learned. The training of the reverse diffusion model typically uses the Variational Lower Bound (VLB) as the loss function, which is formulated as:

(3) $$L=E_{q}\left[\sum_{t=1}^{T}D_{\mathrm{KL}}\left[q\left(x_{t-1}|x_{t},x_{0}\right)\|p_{\theta }\left(x_{t-1}|x_{t}\right)\right]-\log p_{\theta }\left(x_{0}|x_{1}\right)\right]$$

where $D_{\mathrm{KL}}$ represents the Kullback–Leibler divergence, measuring the discrepancy between two distributions.

Although applications in agriculture and forestry are less common, the potential of these models in processing complex ecological data and performing long-sequence predictions has been demonstrated [34]. The lack of extensive ecological and time-series data in forestry production, such as soil data and growth data, necessitates the use of traditional machine learning methods and models of higher complexity. The strength of the fusion model, through simulation of the data process, captures complex relationships and underlying patterns between data more effectively, thereby enhancing the accuracy and effectiveness of data modeling.

In this research, the potential of employing the diffusion model for disease detection and growth assessment in jujube forests was explored, particularly in managing uncertainty and simulating growth processes. Jujube trees, as significant fruit tree saplings, are influenced by various factors such as climate conditions, soil nutrients, and pests. Traditional agricultural predictions, based on experience and simple statistical models, struggle to comprehensively assess the growth condition and health status of jujube forests. By integrating fusion models, the growth processes of jujube trees under different environmental conditions can be simulated, predicting future growth indications.

The application of this fusion model not only explores the possibility for farmers and forestry managers to make scientific planting and management decisions but also provides crucial support for early warning and prevention of pests and diseases [35]. During the growth simulation, the model considers the impacts of different climatic and soil conditions on the growth of jujube trees, with predictive models tailored to specific conditions for types and degrees of pests and diseases. This predictive capability not only aids in timely intervention to reduce losses in forestry production but also optimizes the utilization of resources [36].

### 2.2. Transformer

Since its introduction in 2017, the Transformer architecture has become the preferred model architecture for various tasks, especially when handling complex sequence data [37]. The core of the Transformer is the self-attention mechanism, which processes long-distance dependencies within input data, thereby achieving breakthrough results in fields such as text translation and speech recognition [38]. Attention has also been directed towards the application of Transformers in forestry [39], primarily for parsing remote sensing image data and monitoring tree growth states.

The Transformer model’s key components are the self-attention and multi-head attention mechanisms [40,41]. Self-attention calculates correlations between each element in the input sequence and all other elements, capturing long-distance dependencies. Multi-head attention, by conducting multiple self-attention processes in parallel, enables the model to understand and represent input data from different perspectives and levels [42]. Each head has its own set of query, key, and value vectors and performs independent attention computations, and the outputs from all heads are concatenated and transformed linearly to produce the final output.

In forestry, the application of Transformers is mainly focused on parsing remote sensing image data and monitoring the growth states of trees. Remote sensing technology provides extensive, high-resolution forest imagery [43], and considering rich ecological and environmental factors, such as vegetation cover, land use, and tree growth conditions [44], traditional image processing methods, which rely on manually designed feature extractors and high-resolution algorithms, struggle to fully mine the complex information within image data. The Transformer architecture, through its self-attention mechanism, processes complex relationships between different positions and features in the input data without relying on manually designed feature extractors, offering significant advantages in forestry data analysis and processing [45].

The parsing of remote sensing image data is a critical application of Transformers in forestry [46]. High-resolution image data obtained through remote sensing contain rich geographic and ecological information, such as types of vegetation, coverage, soil moisture, and land-use types [47]. Transformers, through their self-attention mechanism, effectively capture complex relationships among these features, facilitating the analysis of large-scale remote sensing image data. For example, in monitoring tree growth states, Transformers can accurately identify different vegetation types and tree species, assessing vegetation coverage and growth conditions [39]. This provides essential information for forestry managers, aiding in the formulation of scientific management and conservation strategies [48].

Moreover, by actively processing complex relationships between different positions and features within the input database, the Transformer structure, without relying on manually designed extraction tools, provides significant advantages in the analysis and processing of the wood industry. For instance, in monitoring the growth states of trees, Transformers can accurately recognize different vegetation types and tree species and assess vegetation coverage and growth conditions, offering references for forestry managers.

This research further extends the application of the Transformer structure [49], effectively enhancing the model’s efficiency and accuracy in handling large-scale forestry image data through the integration of parallel attention mechanisms. The core idea of parallel attention is to compute multiple self-attention heads in parallel, allowing the model to simultaneously understand and represent input data from different angles and levels [50]. This parallel processing not only improves the model’s computational efficiency but also enhances its ability to capture complex data relationships.

This research aimed to evaluate the utility of diffusion models in detecting diseases and assessing the growth of jujube forests, particularly focusing on managing uncertainties and simulating growth processes under various environmental conditions. Given the importance of jujube trees as fruit-bearing saplings, their growth and health are influenced by multiple factors including climate conditions, soil nutrients, and pest infestations. Traditional methods of agricultural and veterinary predictions, which often rely on empirical knowledge and simplistic statistical models, fall short in providing a comprehensive assessment of the growth conditions and health status of jujube forests. By integrating fusion models, this research sought to simulate the growth processes of jujube trees, thereby enabling predictions of their future growth trajectories under differing environmental scenarios.

## 3. Materials and Method

### 3.1. Materials Collection

**Figure 1:**
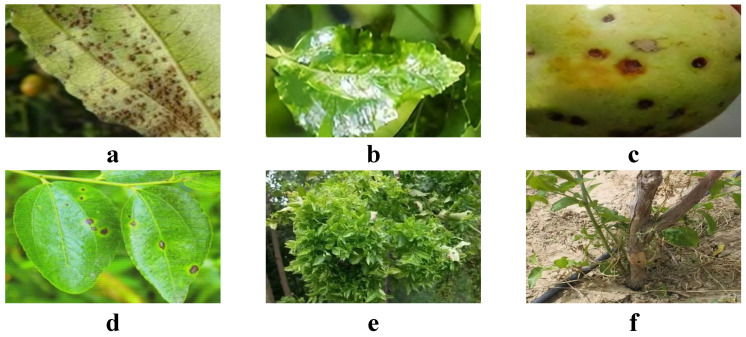
Dataset samples. (**a**) is rust, (**b**) is powdery mildew, (**c**) is anthracnose, (**d**) is black spot disease, (**e**) is date mania, (**f**) is date seedling stem rot.

In this study, data collection was primarily conducted in the Yinshan Mountain Range in Bayannur, Inner Mongolia, focusing on areas such as the sunny slopes at the foot of Wula Mountain and Lang Mountain. These locations, due to their unique geographical and climatic conditions, serve as ideal sites for studying the growth conditions of Chinese jujube. The types and quantities of images collected are shown in Table 1 and Figure 1.

The data collection window was selected to be from March to July 2023, a period that encompasses the full growth cycle of the jujube, from budding to maturity. The sites chosen, such as the sunny slopes at the foot of Wula and Lang Mountains, exhibit good vegetation growth conditions and reflect the typical growth environment and ecological characteristics of the region. Additionally, the climate of the Yinshan Mountain Range falls within the warm temperate to mid-temperate continental climate zone, characterized by dryness and ample sunlight. These climatic conditions not only favor the growth of jujube but also provide a natural experimental setting for studying diseases potentially triggered by climatic factors like drought.

In terms of equipment selection, this study utilized advanced imaging devices and environmental monitoring instruments to ensure the accuracy and reliability of the data. Specifically, high-performance cameras such as the iPhone 13 Pro Max, Canon EOS R10, and Huawei P30 were employed. These devices, known for their high resolution and optimized imaging technologies, capture detailed micro-level plant features and color variations crucial for the early detection and accurate assessment of diseases. Additionally, the Swiss-made Sensirion digital temperature and humidity sensor SHT11 was used to measure environmental parameters. This sensor offers a broad measurement range and high precision, accurately recording temperature and humidity under complex environmental conditions, thus providing reliable data for analyzing plant physiological responses and environmental adaptability.

To comprehensively capture the growth and disease progression of the jujube, multiple fixed monitoring points were established within the study area, and data collection was conducted in spring (March to May) and summer (June to July). This temporal arrangement allowed us to observe various changes in the jujube from budding to maturity and their responses to environmental conditions. At each monitoring point, we ensured the representativeness of vegetation types and the scientific integrity of the samples through pre-analysis of maps and field surveys. Multispectral sensors were also deployed, capturing images in non-visible light ranges, such as near-infrared, to analyze plant health and physiological responses. The use of these devices, combined with regular data collection, enabled us to acquire high-quality, information-rich datasets.

Moreover, to ensure the practicality and scientific validity of the collected image data, a rigorous image inspection and selection process was implemented. In the field, each image was initially screened by the collection team, discarding any that were underexposed or blurry. Subsequently, these pre-screened images were sent to the data processing center where trained technicians conducted more in-depth quality assessments. Regarding plant health conditions, image analysis was employed to identify the physiological and pathological states of the plants. Health assessments were based on leaf color, morphology, size, and growth patterns. For instance, healthy jujube leaves should display a uniform green color, whereas those affected by the disease might show symptoms such as yellow spots, dryness, or deformation. Only those images that met specific standards for clarity, contrast, and color saturation were selected for subsequent model training and analysis tasks.

Furthermore, throughout the research process, an expert team in plant physiology, ecology, and pathology provided essential knowledge and support. They were involved in the in-depth analysis and interpretation of the data and regularly reviewed the data collection and processing procedures to ensure the scientific rigor and quality of the methods.

### 3.2. Dataset Preprocessing

#### 3.2.1. Image Enhancement Based on Traditional Computer Vision Methods

In the data preprocessing phase, image enhancement is a critical step to improve the effectiveness of model training. The goal is to enhance the quality of images, thereby improving the accuracy of feature extraction and the efficiency of model training. Several traditional computer vision techniques were employed to enhance the raw images in this study, including histogram equalization, contrast stretching, and noise reduction.

Histogram equalization is a method used to improve the global contrast of images, particularly effective for images where the background and foreground are either too bright or too dark. By applying this method, a more uniform distribution of brightness levels can be achieved across various regions of the image, thus expanding the overall contrast of the image. The basic principle of histogram equalization can be mathematically expressed with the following formula:

(4) $$s=T\left(r\right)=\left(L-1\right)\int_{0}^{r}p_{r}\left(w\right)dw$$

where *r* and *s* are the pixel values before and after processing, respectively, *L* is the total number of possible intensity levels, and $p_{r}\left(r\right)$ is the normalized histogram, i.e., the probability of the intensity level *r* in the original image.

Contrast stretching, also known as linear stretching, aims to alter the contrast of an image so that the transition from black to white is more pronounced. This method is particularly suited for images with intensity levels concentrated in a small range. Contrast stretching adjusts the lowest and highest input levels and maps them to output levels, thereby enhancing the overall contrast of the image. The transformation formula is given by:

(5) $$s=\frac{\left(r-r_{\min}\right)}{\left(r_{\max}-r_{\min}\right)}\times \left(L-1\right)$$

where $r_{\min}$ and $r_{\max}$ are the minimum and maximum grayscale values in the original image, respectively, and *L* represents the possible maximum grayscale level.

Noise reduction is an indispensable part of image preprocessing, especially since images collected during the acquisition process are often subject to random noise due to environmental influences and equipment limitations. Noise not only reduces image quality but can also significantly impact the results of model training. Common noise reduction techniques include median filtering and Gaussian filtering. Median filtering is a non-linear image processing technique that eliminates noise by replacing each pixel value in the image with the median value from its neighborhood, suitable for removing salt-and-pepper noise. The mathematical expression for median filtering is:

(6) $$s=\mathrm{median}\{r_{k}|k\in \mathrm{Neighborhood}\}$$

Gaussian filtering, on the other hand, uses a Gaussian function as the weighting function for the filter, convolving it with the image to effectively remove Gaussian noise. The convolution kernel of Gaussian filtering is defined as:

(7) $$G\left(x,y\right)=\frac{1}{2\pi \sigma^{2}}e^{-\frac{x^{2}+y^{2}}{2\sigma^{2}}}$$

where σ is the standard deviation of the Gaussian distribution, controlling the width of the filter.

By combining the aforementioned methods, the quality of the image data can be significantly enhanced, thereby providing a solid foundation for subsequent feature extraction and deep learning model training. These image enhancement techniques not only improve the visual effects of the images but also enhance the model’s ability to recognize key information in the images. This is particularly effective in complex forestry scenarios, helping the model accurately identify and analyze the growth conditions and disease situations of trees and providing scientific data support for forestry management.

#### 3.2.2. CutOut, CutMix, Mosaic

**Figure 2:**
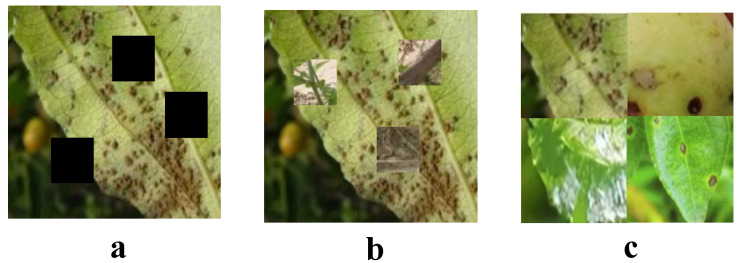
Augmentation methods. (**a**) is Cutout, (**b**) is Cutmix, (**c**) is Mosaic.

In this study, advanced image data augmentation techniques such as CutOut, CutMix, and Mosaic were employed to enhance the training outcomes and predictive accuracy of the model for growth estimation and disease detection in jujube forests, as illustrated in Figure 2. These techniques have significantly improved the model’s ability to recognize disease features in complex forest environments. To overcome the limitations in detailed resolution of drone-captured images, we combined the advantages of high-performance multispectral sensors with ground-based high-resolution images, adopting a multimodal data collection method. Drone images were primarily used for broad-spectrum disease detection and growth assessment, while ground-captured high-resolution images were utilized for precise identification and validation of diseases detected in the drone images. This data collection strategy not only enhanced the coverage and efficiency of disease detection but also ensured the accuracy and reliability of the identifications. Additionally, to train and validate the model effectively, the image data were divided into training, validation, and test sets with specific ratios of 70%, 15%, and 15%, respectively.

CutOut is a straightforward yet effective data augmentation method that enhances model robustness by randomly masking sections of the input images. This forces the model to rely less on dominant features and more on the entirety of the available data, thereby improving generalization. This technique is particularly useful for models that might otherwise focus too much on prominent features at the expense of other informative aspects of the data. In this study, CutOut was utilized to randomly mask portions of jujube tree images, compelling the model to recognize and leverage other relevant features within the images. This enhancement is aimed at improving the model’s sensitivity and accuracy in detecting diseases within the jujube forests. The operation of CutOut can be mathematically represented as follows:

(8) $$I_{\mathrm{new}}=I⊙\left(1-M\right)$$

where *I* represents the original image, and *M* is a mask of the same size as the image, with the masked-out region set to 1 and the rest set to 0, and ⊙ denotes element-wise multiplication.

CutMix, a more complex augmentation technique, not only masks out a portion of an image but also fills this section with a patch from another image. This process compels the model to learn from two different sets of information within a single image during training. In the context of disease detection in jujube forests, CutMix allows the model to learn how to extract key features from complex or incomplete information, enhancing robustness and accuracy in practical applications. The operation of CutMix is defined by the following formula:

(9) $$I_{\mathrm{new}}=M⊙I_{A}+\left(1-M\right)⊙I_{B}$$

where $I_{A}$ and $I_{B}$ are two different input images, and *M* is a randomly generated mask for the region being replaced.

Mosaic is an augmentation technique that stitches together patches from four different images into a single composite image. This method enriches the model’s input by presenting a variety of visual information in a single batch, facilitating the learning of diverse backgrounds and environmental conditions affecting jujube trees. This approach enables the model to adapt more effectively to variations in lighting, backgrounds, and growth conditions of the forests. The concept of Mosaic can foster a more robust learning environment, where the model is trained to handle a wide array of scenarios, thus improving its predictive capabilities across different conditions.

By integrating these three sophisticated image augmentation techniques—CutOut, CutMix, and Mosaic—the study not only significantly enhances the sensitivity and accuracy of the model in detecting diseases within the jujube forests but also bolsters the model’s ability to adapt to complex natural environment variations. The introduction of these techniques provides strong technical support for the early recognition and timely management of diseases in jujube forests, contributing importantly to the improvement of management efficiency and health levels of the forests. This holistic approach to model training and data augmentation ensures that the developed system can perform effectively under diverse operational conditions, ultimately supporting the scientific management and decision-making processes in forestry.

### 3.3. Proposed Method

#### 3.3.1. Overall

**Figure 3:**
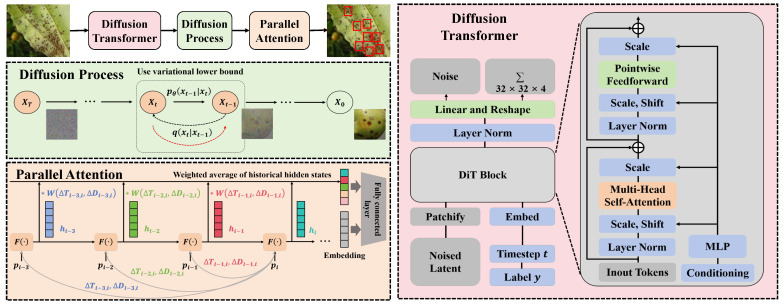
Flowchart of proposed method.

In this study, an advanced deep learning system integrating the Diffusion-Transformer architecture with the parallel attention mechanism is proposed, as shown in Figure 3. The design of this system aims to enhance the precision and efficiency of disease detection through a refined model structure and innovative training strategies. The entire data process from model design and the integration of various modules is detailed below. The core of the system is based on the Diffusion-Transformer architecture, which effectively combines the advantages of generative models with the powerful processing capabilities of the Transformer, making it particularly suitable for handling large-scale and complex image data. Initially, pre-processed image data undergo diffusion model-based data augmentation, generating new images with potential variations, thus providing a richer information base for subsequent disease identification. The diffusion model is primarily used to generate images that are visually similar to the original data but include subtle differences, simulating potential different stages of disease development. Specifically, the diffusion process gradually introduces noise to blur the original images and progressively removes this noise in the reverse process, thereby generating new images. The reverse process is a gradual denoising procedure that incrementally restores clarity to the images. The generated images are then fed into the Transformer model. The Transformer, with its self-attention mechanism, is capable of capturing long-distance dependencies within images, which is crucial for a comprehensive understanding of the image content. In this system, the Transformer is not only used for extracting image features but also for analyzing the interactions among these features, which is highly effective for identifying complex disease patterns. To further enhance the model’s capability to handle specific agricultural image problems, a parallel attention-based agricultural Transformer structure has been specifically designed. This structure employs a multi-head attention mechanism, allowing the model to analyze the image from multiple perspectives, thereby improving its ability to handle complex images. Each head focuses on different aspects of the image; for example, one head may focus on color features, while another may concentrate on texture features. This parallel processing approach not only improves training efficiency but also enables the model to gain a more comprehensive understanding of the image.

During the training process, the parallel loss function is introduced to optimize performance across various aspects, combining classification loss, reconstruction loss, and adversarial loss. The classification loss ensures the model can accurately distinguish between different types of diseases, the reconstruction loss aids the model in learning more image details, and the adversarial loss further enhances the model’s generalization capabilities. The design of this composite loss function allows the model to simultaneously optimize multiple objectives during training, ultimately achieving better learning outcomes. Through this series of designs and configurations, the system not only demonstrates theoretical innovation but also exhibits excellent performance in practical applications, effectively enhancing the accuracy and efficiency of disease detection in jujube forests.

#### 3.3.2. Diffusion-Transformer Architecture

**Figure 4:**
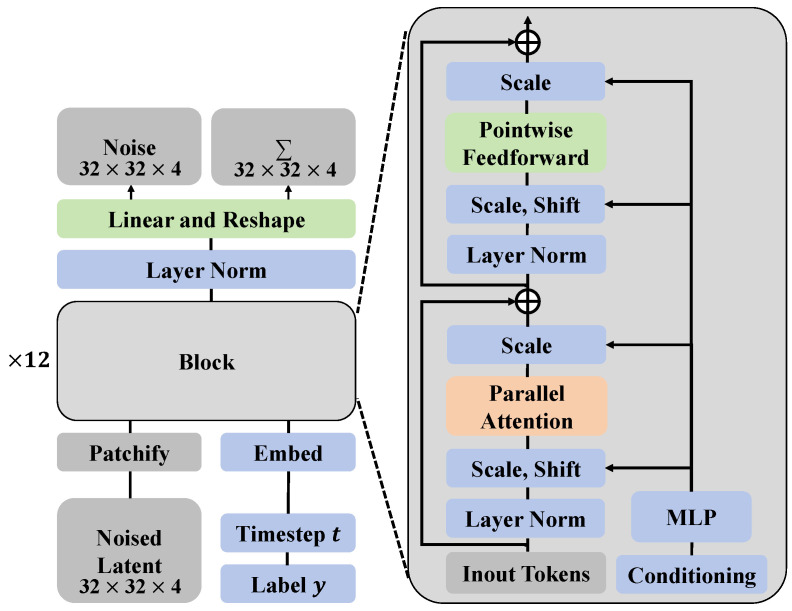
The diagram illustrates a model architecture based on the diffusion transformer, consisting of multiple processing steps and components. Initially, the noise signal undergoes linear transformation and reshaping, followed by layer normalization (Layer Norm). These operations are integrated into multiple repetitive blocks (×12 denotes repetition twelve times).

In this study, an architecture based on Diffusion-Transformer is introduced to effectively assess the growth and detect diseases in jujube forests. This architecture cleverly combines the data generation capability of the diffusion model with the powerful feature extraction ability of the Transformer, tailored for complex and variable forestry image data, as shown in Figure 4. The specific implementation of the Diffusion-Transformer architecture is detailed here, including the model’s network layers, structural design, parameter settings of each layer, and the mathematical analysis and advantages of such a design.

**Figure 5:**
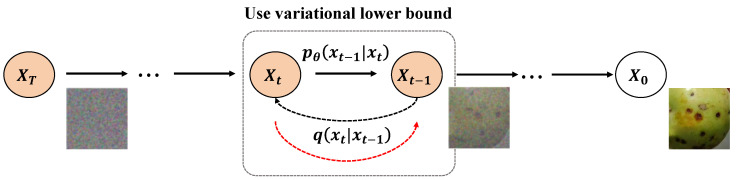
The diagram depicts the application of the diffusion process in image generation, specifically showing how to gradually reverse from the final state $X_{T}$ back to the initial clear state $X_{0}$. The process starts with the noise-added state $X_{T}$, gradually reducing noise through multiple time steps until restoring to the original clear image $X_{0}$. During this process, the variational lower bound is used to optimize each step of the reverse generation, ensuring that each reverse model $q(X_{t-1}|X_{t})$ is as close as possible to the actual forward process $p_{0}\left(X_{t}-1|X_{t}\right)$, which helps to reconstruct the data more accurately and enhance the overall quality of generation. The red dashed lines represent the focus of the model learning in the reverse process, namely how to predict the noise image of the previous state based on the current noise image.

The Diffusion-Transformer architecture consists of two main components: the diffusion model responsible for data generation and the Transformer model responsible for feature extraction. The integration of these two parts allows the system to generate realistic image data and extract useful information for disease detection. The diffusion model is primarily based on a reverse Markov chain, generating new image data by progressively adding and removing noise, as illustrated in Figure 5.

**Figure 6:**
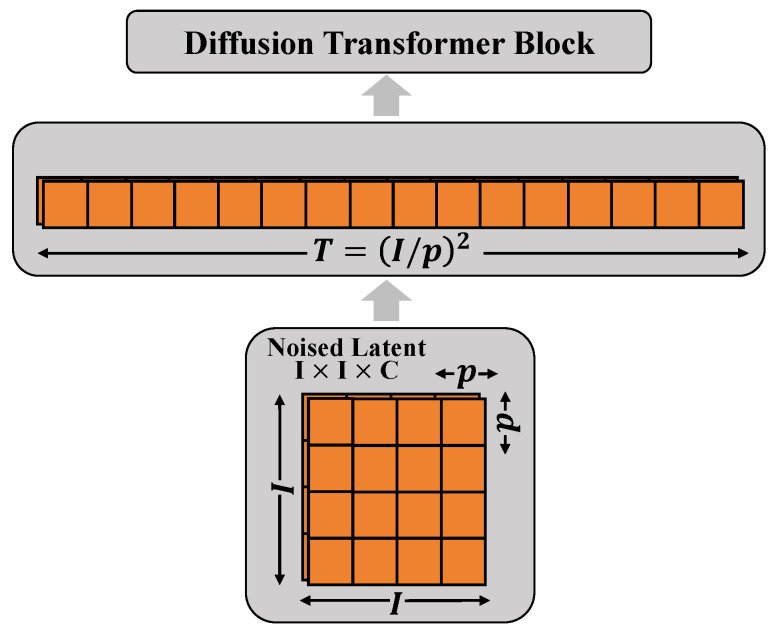
The diagram presents the structural design of the Diffusion Transformer Block. It shows a block consisting of multiple units, each representing a processing step, with the entire block used to handle noised latent feature maps. The dimensions of the latent feature maps are I×I×C, where *I* denotes the height and width of the image, and *C* represents the number of channels. During the diffusion process, each dimension is reduced by a factor of *p*, reflected in the orange squares in the diagram. The Diffusion Transformer Block, by sequentially connecting multiple processing units, incrementally refines the image features, where $T={\left(I/p\right)}^{2}$ represents the total number of processing steps. This design effectively transforms the spatial structure of the image into a sequential form that can be processed by a Transformer network, thus facilitating effective learning of deep features and accurate restoration of image content.

In this system, the diffusion process is designed as a multi-step iterative process, where each step gradually recovers a clear image from the noisy input. Specifically, the diffusion model includes 64 steps, each utilizing a small network composed of convolutional layers to predict the denoised image. Each of these networks has the following parameters: both input and output channels are 128, using a 3 × 3 convolutional kernel, a stride of 1, and no padding to ensure that the image size remains constant throughout the process, as shown in Figure 6.

The Transformer section is designed with multiple layers of parallel attention mechanisms, each including multi-head self-attention units and a feed-forward neural network. The Transformer is configured with 12 layers in this system, each featuring 8 attention heads, allowing the model to capture image features at multiple scales and thereby enhance the understanding of image content. The dimension of each attention head is set to 64, resulting in a total feature dimension of 512 (64 × 8). The feed-forward network uses a two-layer fully connected structure with a ReLU activation function, the first layer having a width of 2048 and the second layer reduced to 512 to match the output of the self-attention layer. The mathematical basis of the diffusion model lies in the process of noise removal from the data, which can be simulated in reverse. Specifically, the denoising process can be represented as:

(10) $$x_{t-1}=\frac{1}{\sqrt{\alpha_{t}}}\left(x_{t}-\frac{1-\alpha_{t}}{\sqrt{1-\alpha_{t}^{2}}}\epsilon_{\theta }\left(x_{t},t\right)\right)$$

where $\alpha_{t}$ is the noise level coefficient, and $\epsilon_{\theta }\left(x_{t},t\right)$ is the noise predicted by the neural network. The integration of the Diffusion-Transformer design enables the model to balance data generation and processing, thus conducting precise disease detection in complex natural environments. The diffusion model enhances model training by generating diverse image data, while the Transformer, with its robust feature extraction capabilities, identifies subtle signs of disease from these data. Moreover, the multi-head self-attention mechanism allows the model to analyze the image from multiple perspectives, improving its comprehension of complex images. The introduction of this architecture significantly enhances the accuracy and efficiency of disease detection, offering substantial value for practical forestry management.

#### 3.3.3. Agricultural Transformer Structure Based on Parallel Attention

**Figure 7:**
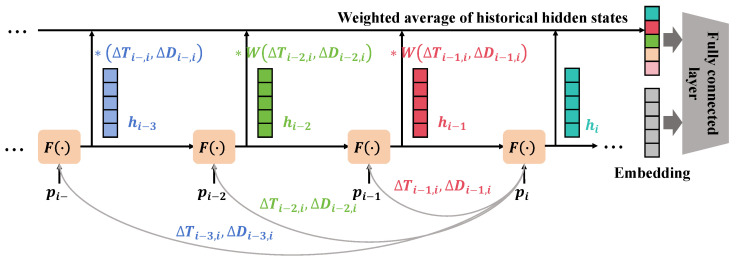
The diagram illustrates how the parallel attention mechanism enhances the model’s capability for time series prediction by computing a weighted average of historical hidden states. In the diagram, various hidden states $h_{i-3}$, $h_{i-2}$, and $h_{i-1}$ represent model outputs at different time steps, each processed by the function F(·). These hidden states are assigned different weights $W(\Delta T_{i-j},\Delta D_{i-j})$, where ΔT and ΔD represent the time interval and data change amount, respectively, used to compute the weighted average, thereby forming the current state $h_{i}$. The * symbol in the diagram represents the element-wise multiplication of the hidden states with the corresponding weights. This process, by reflecting the dynamic changes in historical data, assists the model in making more accurate future state predictions. Finally, $h_{i}$ is further processed through an embedding layer and a fully connected layer, generating the final output.

In this study, an innovative agricultural Transformer structure based on parallel attention is employed to further enhance the precision and efficiency of disease detection and growth assessment in jujube forests, as illustrated in Figure 7. This structure optimizes the model’s capability to capture agricultural image features by processing various types of attention mechanisms in parallel. Traditional Transformer self-attention mechanisms calculate the weight distribution among input features, focusing on important parts of the input to enhance the model’s expressive power. While powerful across many domains, a single attention mechanism may not fully capture the diverse information in complex agricultural images, such as different growth stages of plants or early signs of disease. To address this issue, the parallel attention mechanism was designed, incorporating multiple parallel attention modules into the existing Transformer architecture. Each module focuses on capturing different features in the image (such as color, texture, shape) and integrates this information to achieve a more comprehensive understanding of the image. This design ensures that each type of information is treated equally and effectively expressed within the model.

The core of parallel attention lies in the parallel computation of outputs from multiple self-attention layers, subsequently integrating these outputs to enhance the model’s representational ability. Specifically, with *N* parallel attention layers, the output of each layer is:

(11) $${\mathrm{Attention}}_{i}\left(Q,K,V\right)=\mathrm{softmax}\left(\frac{Q_{i}K_{i}^{T}}{\sqrt{d_{k}}}\right)V_{i},\;i=1,2,\ldots ,N$$

Here, $Q_{i}$, $K_{i}$, and $V_{i}$ represent the query, key, and value matrices for the *i*th attention head, respectively, and $d_{k}$ is the dimension of the key. The outputs from these parallel attention layers are then integrated by a fusion function *F*:

(12) $$\mathrm{PA}-\mathrm{Out}=F({\mathrm{Attention}}_{1},{\mathrm{Attention}}_{2},\ldots ,{\mathrm{Attention}}_{N})$$

In this context, the fusion function is an operation of weighted summation, which presents several advantages for using parallel attention:

1. Handling of Information Diversity: Agricultural images typically contain rich information, such as variations in color or leaf textures, crucial for disease detection. Parallel attention allows for a comprehensive understanding of the image content by processing these different types of information in parallel.
2. Enhanced Expressive Capability of the Model: By processing various attention mechanisms in parallel, the model significantly enhances its ability to capture key features without adding extra complexity, thus improving the accuracy and efficiency of disease detection.
3. Strong Adaptability: This mechanism permits the model to adjust the importance of each attention layer according to the demands of different tasks, enhancing the model’s adaptability and flexibility.

The introduction of parallel attention in the task of disease detection and growth analysis in jujube forests significantly enhances the model’s ability to recognize early signs of disease, especially when disease characteristics are not pronounced. The integration of multi-angle information allows the model to detect signs of disease from subtle changes, greatly enhancing the practicality and scientific validity of the model.

#### 3.3.4. Parallel Loss Function

In the system developed for assessing the growth and detecting diseases in jujube forests using the Diffusion-Transformer architecture, an innovative loss function, known as the parallel loss function, has been designed. This loss function significantly differs from those traditionally employed in Transformer models and is optimized for the specific tasks of this study. The parallel loss function is conceived to address the challenges of multi-objective optimization in complex tasks, integrating various loss components to simultaneously optimize multiple aspects of the model. Specifically, this loss function comprises three main parts: classification loss, reconstruction loss, and adversarial loss, each targeting a specific training objective:

1. Classification Loss ($L_{cls}$): optimizes the model’s performance in disease detection tasks, specifically the accurate classification of diseased and healthy states within images.
2. Reconstruction Loss ($L_{recon}$): aims to enhance the model’s ability to learn detailed features of the input data through a reconstruction task, forcing the model to capture more details and improve its understanding of images.
3. Adversarial Loss ($L_{adv}$): enhances the model’s generalization capabilities by incorporating ideas from generative adversarial networks, ensuring stability and high performance on generated data.

The overall formula for the parallel loss function is expressed as:

(13) $$L_{total}=\lambda_{cls}L_{cls}+\lambda_{recon}L_{recon}+\lambda_{adv}L_{adv}$$

where $\lambda_{cls}$, $\lambda_{recon}$, and $\lambda_{adv}$ are the weight coefficients for each loss component, adjusted according to the specific needs of the task. Traditional Transformer models typically employ a cross-entropy loss function for training in classification or sequence prediction tasks. Although this single loss function performs well in many tasks, it may not be sufficient for complex tasks that require the simultaneous optimization of multiple objectives. For instance, in disease detection tasks, optimizing solely for classification accuracy might neglect the model’s learning of image details, crucial for identifying early or minor disease symptoms. The use of the parallel loss function enables the model to focus on multiple critical performance metrics during training, such as classification accuracy, the quality of image reconstruction, and model generalization. This is because the parallel loss function offers mathematical advantages in joint optimization of the loss functions. For a given set of training data (x,y), where *x* is the input image and *y* is the corresponding label, the model aims to minimize the total loss $L_{total}$.

In applications for disease detection and growth analysis in jujube forests, the parallel loss function demonstrates several advantages:

1. Enhanced Detection Precision: By optimizing both classification and reconstruction losses, the model not only accurately identifies the presence of diseases but also better understands subtle differences in images, crucial for early disease detection.
2. Increased Model Robustness: The inclusion of adversarial loss maintains the model’s discriminative ability when confronted with new disease types or variations not covered in the dataset. This enhanced generalization capability, achieved through adversarial training, is vital for dealing with the diverse disease conditions often encountered in practical applications.
3. Improved Detail Capturing: The reconstruction loss compels the model to focus on reconstructing the input image, which not only helps the model learn more image details but also enhances its understanding of the overall image structure. This is particularly effective in handling complex forestry images, helping the model identify subtle but critical signs of disease.
4. Controllable Training Process: By adjusting the weight coefficients ($\lambda_{cls}$, $\lambda_{recon}$, $\lambda_{adv}$) of each loss function, the training process can be flexibly controlled to optimize each objective to the extent required by specific application needs, thus better adapting to the complex and variable real-world environments.

### 3.4. Evaluation Metrics

This study employs four key metrics to evaluate the performance of the Diffusion-Transformer architecture: Precision, Recall, Accuracy, and F1-score. Precision calculates the proportion of true positive predictions among all positive predictions, serving as a measure of the accuracy of positive predictions. Recall indicates the model’s ability to identify all relevant cases, measuring the completeness of positive identifications. Accuracy reflects the overall correctness of the model’s predictions, encompassing both positive and negative instances. Lastly, the F1-score, which is the harmonic mean of Precision and Recall, provides a balance between them, especially useful in scenarios with unbalanced datasets. Together, these metrics allow for a comprehensive evaluation of the model’s performance, guiding further optimization and adjustments.

By considering these evaluation metrics comprehensively, the performance of the Diffusion-Transformer-based system for disease detection in jujube forests can be thoroughly analyzed and assessed. The application of these metrics, combined with experimental data and feedback from field applications, will further promote the development of intelligent forestry management systems, enhancing their efficiency and accuracy in practical operations.

### 3.5. Baseline

To comprehensively evaluate the performance of the growth assessment and disease detection system in jujube forests based on the Diffusion-Transformer architecture, several baseline models were employed for comparative analysis. These models include traditional machine learning algorithms such as Support Vector Machines (SVMs) [51] and Random Forest [52], along with deep learning architectures like AlexNet [53], ResNet [54], Vision Transformer (ViT) [26], and EfficientNet [55]. Each model has distinct features, and their performance comparisons validate the proposed system’s advantages and applicability comprehensively. By comparing the performance of the Diffusion-Transformer-based system with these classical and advanced machine learning and deep learning models, the capabilities of the new system in processing complex forestry image data are thoroughly evaluated. This comparison not only highlights the unique advantages of the new model but also identifies areas for further optimization, providing valuable insights for future model improvements and applications.

### 3.6. Experimental Setup

To comprehensively evaluate the performance of the system based on the Diffusion-Transformer architecture for assessing growth and detecting diseases in jujube forests, a series of experimental setups were meticulously designed, encompassing hardware configuration, software setup, training strategy, and hyperparameter settings. These configurations ensured the efficiency and scientific integrity of the experiments, providing a robust foundation for the model’s application in actual forestry management.

In terms of hardware configuration, considering the high computational demands of deep learning, especially models incorporating large Transformers, a computing cluster equipped with high-performance GPUs was utilized. Each node was equipped with NVIDIA Tesla V100 GPUs, each having 32 GB of memory, which provided substantial computational power for handling complex model structures and large datasets. Additionally, high-speed Ethernet connections between compute nodes ensured efficient data transfer, significantly accelerating the training process and data handling. For software setup, the experiments were conducted on a Linux operating system, with Python selected as the primary programming language due to its extensive libraries for scientific computing and machine learning. NumPy and Pandas were utilized for data handling, while Matplotlib and Seaborn facilitated data visualization. The training and testing of deep learning models relied on the PyTorch framework, which offers flexible and powerful tools for building and training complex models, as well as supporting automatic differentiation that greatly simplifies the development of complex architectures. The OpenCV library was employed for a range of image preprocessing tasks such as cropping, rotating, and scaling, ensuring standardization and quality of input data.

Regarding training strategies, five-fold cross-validation was employed to assess the model’s generalization ability and stability. Specifically, the dataset was randomly divided into five exclusive subsets, with four subsets used for training and the remaining subset used for validation in each training phase. This process was repeated five times, rotating the validation subset each time, ensuring that each data point was used for validation once, effectively reducing the randomness in model evaluation and enhancing the reliability of the assessments. Hyperparameter settings were determined using a grid search method, adjusting parameters such as learning rate, batch size, and training epochs based on performance feedback from the validation set. The initial learning rate was set at 0.001, with an exponential decay strategy reducing it by 10% every 10 epochs to facilitate rapid convergence initially and more detailed adjustment later. Batch sizes were typically set at 64, adjusted according to the complexity of the model and memory constraints. The total training period was set to 100 epochs to ensure thorough learning from the data features. To prevent overfitting, Dropout and L2 regularization techniques were implemented during training, randomly deactivating neurons within the network and penalizing weights to enhance the model’s generalization capabilities for unseen data.

## 4. Results and Discussion

### 4.1. Disease Detection Experimental Results

**Figure 8:**
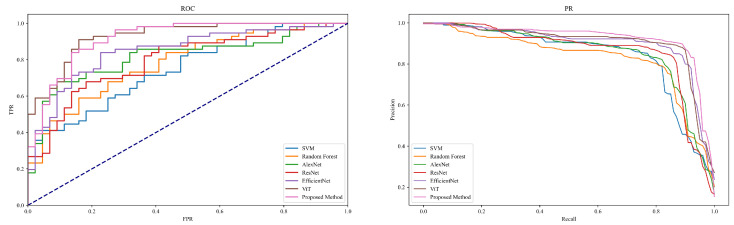
ROC and PR curves of Table 2.

This section is dedicated to evaluating and comparing the performance of different models in the task of disease detection within jujube forests, aimed at validating the effectiveness of the Diffusion-Transformer-based approach in practical applications. The performance of each model was quantified using four core metrics, Precision, Recall, Accuracy, and F1-score, to thoroughly assess their capabilities in disease identification, as shown in Table 2 and Figure 8.

The experimental results reveal a progressive improvement in model performance, reflecting the gradual enhancements from traditional machine learning to modern deep learning models. The SVM, as a basic machine learning model, showed relatively weaker performance in the disease detection task with a precision of 0.83, recall of 0.80, accuracy of 0.81, and an F1-score of 0.82. The Random Forest, an ensemble learning model that builds multiple decision trees and aggregates their predictions to enhance performance, demonstrated a precision of 0.85, recall of 0.82, accuracy of 0.83, and an F1-score of 0.84. These models, although effective in handling simple or structured data, are limited in their performance on high-dimensional and complex image data due to their feature extraction and handling capabilities. Deep learning models like AlexNet, ResNet, and EfficientNet exhibited higher performance. AlexNet, as an early deep convolutional neural network, leverages multiple layers for feature extraction and learning, achieving a precision of 0.87, recall of 0.84, accuracy of 0.85, and an F1-score of 0.86. ResNet addresses the training challenges of deep networks by introducing residual connections, allowing deeper image feature learning with a precision of 0.89, recall of 0.86, accuracy of 0.87, and an F1-score of 0.88. EfficientNet balances performance by adjusting network depth, width, and resolution, achieving precision of 0.91, recall of 0.88, accuracy of 0.89, and an F1-score of 0.90. These models, through more complex architectural designs and optimized training strategies, effectively extract and utilize complex information from images, thus enhancing the accuracy of disease detection. The ViT and the method proposed in this study showed the best performance among all models. ViT applies the Transformer structure to image analysis, capturing the global relationships between image blocks through its self-attention mechanism, which resulted in precision of 0.93, recall of 0.90, accuracy of 0.91, and an F1-score of 0.92. The method introduced in this study, which combines the data augmentation capabilities of generative models with the efficient feature processing mechanisms of Transformers, further enhanced performance, achieving precision of 0.95, recall of 0.92, accuracy of 0.93, and an F1-score of 0.94. This success is largely attributed to the Diffusion-Transformer structure’s ability to generate realistic variant image data to enrich the training set and its meticulous feature extraction process, effectively capturing disease characteristics. In summary, the experimental results confirm the incremental improvements in disease detection from traditional machine learning to deep learning and innovative Transformer models, particularly the novel architecture proposed in this study which significantly enhances both the accuracy and efficiency of disease detection. These findings not only demonstrate the potential of these models in practical applications but also provide crucial references for future model selection and optimization.

### 4.2. Jujube Forest Growth Estimation Experimental Results

**Figure 9:**
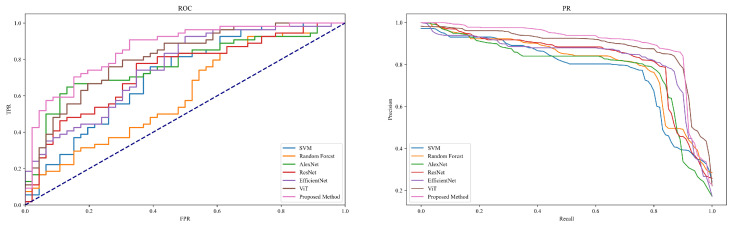
ROC and PR curves of Table 3.

This study aims to validate the practical effectiveness of the Diffusion-Transformer-based system for growth estimation and disease detection in jujube forests. The performance of the models is quantified using four key indicators: Precision, Recall, Accuracy, and F1-score. The experimental design includes a variety of benchmark models encompassing both traditional machine learning and deep learning models, compared against the method proposed in this paper to demonstrate its advantages in growth estimation, as shown in Table 3 and Figure 9. This approach allows for a comprehensive analysis and evaluation of the performance of each model in practical applications, thereby confirming the effectiveness and scientific validity of the proposed method.

The experimental results show that traditional machine learning models like the SVM and Random Forest exhibit lower accuracy and F1-scores. SVM achieved a precision of 0.80, recall of 0.77, accuracy of 0.79, and an F1-score of 0.78, while Random Forest recorded a precision of 0.82, recall of 0.79, accuracy of 0.81, and an F1-score of 0.80. Although these models perform well on datasets with fewer features or less complexity, their effectiveness is limited when handling complex forestry image data due to their feature extraction and processing capabilities. In contrast, deep learning models such as AlexNet, ResNet, and EfficientNet demonstrate higher performance. AlexNet, with a precision of 0.84, recall of 0.81, accuracy of 0.83, and an F1-score of 0.82, utilizes deep convolutional layers to effectively extract and learn complex features. ResNet, enhancing the learning depth with residual connections, shows a precision of 0.86, recall of 0.83, accuracy of 0.85, and an F1-score of 0.84. EfficientNet, which balances network dimensions for optimized performance, achieves a precision of 0.88, recall of 0.85, accuracy of 0.87, and an F1-score of 0.86. The ViT and the method introduced in this study show the highest performance. ViT, employing self-attention to capture global relationships between image segments, attains a precision of 0.90, recall of 0.87, accuracy of 0.89, and an F1-score of 0.88. The proposed method further enhances performance by combining generative model capabilities with efficient Transformer mechanisms, achieving precision of 0.92, recall of 0.89, accuracy of 0.91, and an F1-score of 0.90. These significant improvements are attributed to the innovative model design, particularly effective in handling long-sequence data and high-dimensional features. The combination of generative models and Transformers allows the system to perform feature extraction and disease detection effectively, greatly improving the accuracy and efficiency of growth estimation. Overall, the experimental results not only demonstrate the advantages of deep learning models over traditional machine learning approaches in the task of growth estimation but also highlight the significant performance enhancements achieved by the proposed method in handling complex forestry image data. These findings provide a robust theoretical and empirical foundation for further model optimization and practical application.

### 4.3. Ablation Study on Different Attention Mechanisms

**Figure 10:**
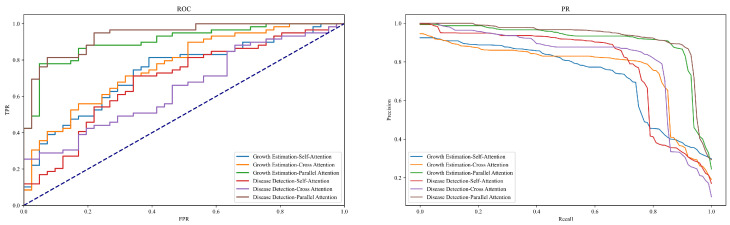
ROC and PR curves of Table 4.

The purpose of this section’s experiment is to explore the impact and effectiveness of various types of attention mechanisms in the tasks of growth estimation and disease detection in jujube forests through ablation studies. The experimental design includes three different attention mechanisms: Self-Attention, Cross Attention, and Parallel Attention. These mechanisms were applied to both growth estimation and disease detection tasks to assess their performance, thereby revealing the specific contributions of different attention mechanisms to model recognition capabilities, as shown in Table 4 and Figure 10.

The results indicate that regardless of the task, whether growth estimation or disease detection, the parallel attention mechanism demonstrates superior performance, followed by cross attention, with self-attention showing relatively weaker results. Specifically, in the growth estimation task, the model using parallel attention achieved precision, recall, accuracy, and F1-score of 0.92, 0.89, 0.91, and 0.90, respectively, significantly higher than the results using self-attention and cross attention. Similarly, in the disease detection task, the parallel attention mechanism led with a precision of 0.95, recall of 0.92, accuracy of 0.93, and F1-score of 0.94, outperforming the other two mechanisms.

Theoretical analysis suggests that while the self-attention mechanism can capture global dependencies within a sequence by computing the relationships among elements, it may not fully utilize local features when dealing with images possessing complex spatial characteristics. This mechanism performs adequately in basic scenarios of growth estimation and disease detection but falls short in tasks requiring more detailed image feature analysis. The cross attention mechanism, by introducing an additional flow of information (such as data from different modalities or features from different processing stages), enhances the model’s focus on critical information, thus offering an improvement over self-attention in complex tasks. This mechanism, through cross-learning from different data sources, better grasps the relationship between disease features and plant growth states, thereby enhancing recognition precision. The significant advantage of the parallel attention mechanism stems from its ability to process multiple types of attention computations simultaneously. This parallel processing approach not only increases the width of information processing by the model but also optimizes depth. Each attention head independently focuses on different aspects of the input data, such as texture, shape, color, etc., and then integrates these independently processed pieces of information to form a comprehensive, multidimensional understanding of the data. This mechanism is particularly suitable for processing images from jujube forests, which possess rich biological and environmental features, effectively enhancing the model’s sensitivity to subtle pathologies and its adaptability to complex backgrounds.

### 4.4. Ablation Experiment on Different Loss Functions

**Figure 11:**
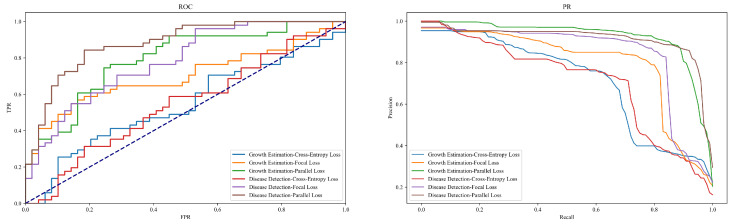
ROC and PR curves of Table 5.

The objective of this section’s experiment is to evaluate and compare the performance and impact of different loss functions in the tasks of growth estimation and disease detection in jujube forests. The experimental setup involved a comparison among three types of loss functions: the traditional Cross-Entropy Loss, the Focal Loss designed for class imbalance, and the Parallel Loss function proposed in this study. This ablation experiment aims to reveal the specific impacts of different loss functions on model performance, particularly in handling agricultural image data with varying difficulties and features, as shown in Table 5 and Figure 11.

The results demonstrate that models employing the parallel loss function consistently exhibit the highest precision, recall, accuracy, and F1-score across both tasks. Specifically, in the growth estimation task, the parallel loss achieved a precision of 0.92, recall of 0.89, accuracy of 0.91, and an F1-score of 0.90; in the disease detection task, this loss function achieved even more impressive scores, with a precision of 0.95, recall of 0.92, accuracy of 0.93, and an F1-score of 0.94. In contrast, models using traditional cross-entropy loss exhibited significantly lower performance in both tasks, with precision scores of 0.70 and 0.73, respectively, indicating that a single loss function might struggle to drive sufficient learning of complex features in intricate agricultural image analysis tasks.

Theoretical analysis suggests that while traditional cross-entropy loss functions perform well in many standard machine learning tasks, they may fall short in agricultural image data scenarios characterized by class imbalance or ambiguous labels due to their simplistic nature, which may not adequately handle complex or subtle data features. The focal loss, an enhanced loss function, adjusts the focus parameter within the loss to address class imbalance by increasing the penalty on hard-to-classify samples, thereby improving the model’s recognition of minority classes. This characteristic significantly elevates its performance in growth estimation and disease detection tasks compared to cross-entropy loss. The design of the parallel loss function takes into account multiple aspects: precision in classification, the model’s ability to capture details, and robustness against adversarial examples. This loss function combines classification loss, reconstruction loss, and adversarial loss, not only emphasizing high-precision predictions for correct categories but also encouraging the model to focus on reconstructing details of the input data, enhancing the model’s sensitivity and analytical capabilities towards input details, which are crucial for early detection of diseases. Additionally, the introduction of adversarial loss enhances the model’s adaptability to variations in input, maintaining high accuracy across different environmental conditions. This multifaceted approach significantly boosts model performance, particularly in complex agricultural imaging tasks, making the parallel loss function especially effective for early disease detection and robust across varied imaging conditions.

### 4.5. Future Work

Although this study has achieved significant success in the growth estimation and disease detection of jujube forests, there are still some limitations and future research directions. First, in terms of data collection, although drone photography and various sensors were used to ensure the diversity and coverage of the data, the complexity of the forest environment and the variations under different seasonal and climatic conditions may affect the consistency and representativeness of the data. Moreover, the dataset is mainly concentrated in the experimental station in Bayannur, Inner Mongolia, which may not fully cover the diversity of jujube forests across the country. Therefore, the generalizability of the model needs to be validated in a broader geographical area. Secondly, in terms of model design, although the parallel attention and Diffusion-Transformer structures have shown excellent performance, these models consume substantial computational resources, particularly in applications requiring real-time or near-real-time processing of large data volumes, where computational delays and resource demands may become limiting factors. Additionally, while multiple loss functions were introduced for comprehensive optimization, improving the model’s performance in complex tasks, the setting of loss function weights relies on empirical adjustment and may require a more scientific method to dynamically adjust these parameters to suit different task requirements and data characteristics. Furthermore, although the experiments covered a variety of models and technologies and verified the effectiveness of the proposed methods, the model still needs to face more complex and dynamically changing environments in practical applications. For instance, new types of diseases in jujube forests, sudden disease events, and interactions between different diseases all require the model to have higher adaptability and flexibility.

In response to these limitations, future research work can be expanded and deepened in several areas. First, expand the scope and depth of data collection by including data from different regions and types of forests, especially those in areas with extremely different ecological conditions, to enhance the model’s generalizability and adaptability. Second, optimize the model structure and training strategies by exploring more efficient model architectures or reducing the computational demands through techniques such as model compression and quantization, making it more suitable for deployment in resource-limited environments. Additionally, develop smarter strategies for adjusting the weights of loss functions, possibly by introducing methods such as reinforcement learning to dynamically adjust the weights of various parts of the loss function to adaptively optimize model performance.

## 5. Conclusions

This study aims to develop an efficient system for detecting diseases in jujube forests and to enhance the accuracy of disease identification by integrating advanced deep learning techniques and graph attention mechanisms. Through a series of experiments, we validated the effectiveness of the proposed model, particularly its outstanding performance in detecting various complex diseases. The experimental results demonstrate that the proposed method significantly outperforms existing approaches across key metrics, especially in terms of precision (0.94), recall (0.92), and accuracy (0.93). Moreover, by comparing different attention mechanisms and loss functions, this study further validates the effectiveness of the graph attention mechanism and optimized loss functions in improving model performance. The introduction of these innovative techniques not only enhances the accuracy of disease detection but also improves the model’s ability to handle complex agricultural data, showcasing its broad potential for practical applications. In conclusion, this study successfully applies advanced deep learning techniques to the field of smart agriculture, achieving significant progress in the detection of diseases in jujube forests. Future research will continue to optimize the model structure, improving the system’s practicality and scalability and providing stronger technical support for the development of smart agriculture.

## Figures and Tables

**Table 1:** Dataset distribution.

Disease Type	Quantity
Powdery Mildew	897
Black Spot Disease	901
Anthracnose	1082
Rust	1169
Date Mania	1043
Date Seedling Stem Rot	965

**Table 2:** Disease detection results comparison.

Model	Precision	Recall	Accuracy	F1-Score
SVM	0.83	0.80	0.81	0.82
Random Forest	0.85	0.82	0.83	0.84
AlexNet	0.87	0.84	0.85	0.86
ResNet	0.89	0.86	0.87	0.88
EfficientNet	0.91	0.88	0.89	0.90
ViT	0.93	0.90	0.91	0.92
Proposed Method	0.95	0.92	0.93	0.94

**Table 3:** Growth estimation results comparison.

Model	Precision	Recall	Accuracy	F1-Score
SVM [12]	0.80	0.77	0.79	0.78
Random Forest [13]	0.82	0.79	0.81	0.80
AlexNet [15]	0.84	0.81	0.83	0.82
ResNet [16]	0.86	0.83	0.85	0.84
EfficientNet [23]	0.88	0.85	0.87	0.86
ViT [28]	0.90	0.87	0.89	0.88
Proposed Method	0.92	0.89	0.91	0.90

**Table 4:** Ablation experiment on different attention mechanisms.

Model	Precision	Recall	Accuracy	F1-Score
Growth Estimation-Self-Attention	0.76	0.73	0.74	0.75
Growth Estimation-Cross Attention	0.83	0.80	0.81	0.82
Growth Estimation-Parallel Attention	0.92	0.89	0.91	0.90
Disease Detection-Self-Attention	0.79	0.75	0.77	0.77
Disease Detection-Cross Attention	0.87	0.82	0.85	0.84
Disease Detection-Parallel Attention	0.95	0.92	0.93	0.94

**Table 5:** Ablation experiment on different loss functions.

Model	Precision	Recall	Accuracy	F1-Score
Growth Estimation-Cross-Entropy Loss	0.70	0.67	0.68	0.68
Growth Estimation-Focal Loss	0.85	0.80	0.83	0.84
Growth Estimation-Parallel Loss	0.92	0.89	0.91	0.90
Disease Detection-Cross-Entropy Loss	0.73	0.70	0.71	0.72
Disease Detection-Focal Loss	0.87	0.83	0.85	0.85
Disease Detection-Parallel Loss	0.95	0.92	0.93	0.94

## Data Availability

The original contributions presented in the study are included in the article, further inquiries can be directed to the corresponding author.
